# Bioinformatic analysis of the RNA expression patterns in microgravity-induced bone loss

**DOI:** 10.3389/fgene.2022.985025

**Published:** 2022-11-08

**Authors:** Xiaoyan Zhang, Tong Xue, Zebing Hu, Xian Guo, Gaozhi Li, Yixuan Wang, Lijun Zhang, Liqun Xu, Xinsheng Cao, Shu Zhang, Fei Shi, Ke Wang

**Affiliations:** The Key Laboratory of Aerospace Medicine, Ministry of Education, Air Force Medical University, Xi’an, China

**Keywords:** bioinformatics, transcriptome, microgravity, bone loss, miRNA

## Abstract

Researchers have linked microgravity in space to the significant imbalance between bone formation and bone resorption that induces persistent bone loss in load-bearing bones. However, the underlying molecular mechanisms are still unclear, which hinders the development of therapeutic measures. The aim of this study was to identify hub genes and explore novel molecular mechanisms underlying microgravity-induced bone loss using transcriptome datasets obtained from the GEO and SRA databases. In summary, comparative RNA expression pattern studies that differ in species (*Homo* or *Mus*), models (*in vitro* or *in vivo*), microgravity conditions (real microgravity or ground-based simulators) and microgravity duration showed that it is difficult to reach a consistent conclusion about the pathogenesis of microgravity-induced bone loss across these studies. Even so, we identified 11 hub genes and some miRNA-mRNA interactions mainly based on the GSE100930 dataset. Also, the expression of CCL2, ICAM1, IGF1, miR-101-3p and miR-451a markedly changed under clinorotation-microgravity condition. Remarkedly, ICAM1 and miR-451a were key mediators of the osteogenesis of hMSCs under clinorotation-microgravity condition. These findings provide novel insights into the molecular mechanisms of bone loss during microgravity and could indicate potential targets for further countermeasures against this condition.

## Introduction

Long-term exposure to spaceflight significantly disturbs the physiological balance of humans. Microgravity, electromagnetic radiation, high vacuum, low magnetic field, ultralow temperature, long-term isolation and limited activities in space have great impacts on human health. Among them, microgravity has the most typical influences on humans and has attracted global attention in the field of aerospace medicine. Studies have shown that long-term exposure to microgravity dramatically disrupts the balance between bone resorption and bone formation, leading to irreversible bone loss (Lang et al., 2004). During a 4- to 6-month spaceflight mission, the bone mineral density (BMD) in the load-bearing bones of astronauts is lost at a rate of 0.5%–1.5% per month and struggles to return to the normal level on the ground (LeBlanc et al., 2000). Microgravity-induced bone loss increases the risk of a variety of diseases, such as bone fracture, cardiovascular calcification and urinary calculi, which has been limiting the development of manned space flight. Unfortunately, the adopted protective measures have not effectively prevented the occurrence of microgravity-induced bone loss thus far. Therefore, it is a priority to reveal the mechanisms of microgravity-induced bone loss at the molecular level to find an ideal therapeutic target.

Due to the huge cost of spaceflight, it is extremely impractical for most researchers to conduct studies in real microgravity environments; thus, it is necessary to establish effective ground-based microgravity models (Patel et al., 2007). Many *in vivo* and *in vitro* microgravity simulators, including the −6° head-down-tilt bed rest (HDBR) (Pavy-Le Traon et al., 2007), hindlimb unloading (HLU) (Morey et al., 1979), random positioning machine (RPM) (Hoson et al., 1997), rotary cell culture system (RCCS) (Duray et al., 1997), rotating wall vessel (RWV) (Schwarz et al., 1992) and two-dimensional (2-D) clinostats (Dedolph et al., 1967), are widely used to partly simulate the effects of microgravity. However, the mechanism of each method is unique. Additionally, bone research under microgravity conditions can be performed through various *in vitro* and *in vivo* models from different origins (Vico et al., 2000; Dai et al., 2007; Swift et al., 2010). Generally, the available cell models *in vitro* include primary bone marrow mesenchymal stem cells (BM-MSCs), primary trabecular and calvarial osteoblasts (TOs and COs), and the MG-63, 2T3 and MC3T3-E1 cell lines. Compared with cell lines, primary cells have obvious advantages of encompassing biological processes that are more reflective of those *in vivo*. However, they are hard to obtain and culture because of their limited lifespan. In contrast, cell lines are more often applied because they can provide a more homogenous population of cells and more reliable reproducibility (Czekanska et al., 2012). *In vivo*, the blood and load-bearing bones are often used to elucidate the biological phenomenon and molecular mechanism underlying microgravity-induced bone loss (Caillot-Augusseau et al., 1998; Lee et al., 2020).

The initiation of the Human Genome Project has given rise to the broad application of transcriptomic strategies that are low-cost, highly efficient and simple to operate, and they promote the arrival of the metagenomic era. Microarray and RNA sequencing (RNA-Seq) are two dominant contemporary techniques extensively used for transcriptome profiling. Microarrays can quantify a set of predetermined probes to detect the expression of defined transcripts, while RNA-Seq captures all sequences by using high-throughput sequencing (Schena et al., 1995). As a consequence, transcriptomic analysis has enabled us to provide information on how genes are regulated in different conditions and has offered a good way to better understand the mechanisms of human disease. Without exception, a range of transcriptomic analyses were performed to explore the mechanisms of microgravity-induced bone loss, and many novel results were demonstrated (Supplementary Table S1). However, it is becoming increasingly urgent that comparisons should be made between these studies that vary widely in species, models and duration.

In this study, we selected transcriptome data from humans and mice associated with microgravity-induced bone loss in the GEO and SRA databases. After data processing, we compared DEGs and enrichment analysis results between different studies from humans or mice to identify common genes and biological phenomena. For the human data, we first identified 11 hub genes and some miRNA-mRNA interactions in real microgravity mainly based on the GSE100930 dataset and validated that CCL2, ICAM1, IGF1, miR-101-3p and miR-451a were clinorotation-sensitive and ICAM1 and miR-451a were osteogenesis mediators under clinorotation-microgravity condition. Additionally, the RPM data in the GSE114117 dataset shared 22 genes and some potential mechanisms with the results in the GSE100930 dataset. For the mouse data, enrichment and PPI analysis of the SRP276872 dataset illustrated that some hub genes, potential biological functions and signaling pathways were involved in microgravity-induced bone loss. Unexpectedly, three genes and some potential mechanisms that showed the same performance in RPM, RWV and RCCS did not interact with the results in real microgravity conditions. In summary, our study provides an overall review of the transcriptome data about microgravity-induced bone loss and elucidated the similarities and differences between these studies by bioinformatics in the hope of finding novel mechanisms and guiding researchers to choose appropriate methods to conduct studies on microgravity-induced bone loss.

## Methods

### Acquisition of transcriptome data

The GEO and SRA databases were used to obtain transcriptome data about microgravity-induced bone loss with the query “microgravity AND bone”. After further screening, we selected eight studies as our study datasets (Supplementary Table S1).

### Raw data processing and identification of DEGs

The original expression matrix of the microarray downloaded from the GEO database by the R package GEOquery was normalized by the “Limma” package in R software (Ritchie et al., 2015). After ID conversion and the duplicated gene symbols were removed, PCA was used to detect the differences between different groups. Then, DEGs were screened out by “Limma” with a *p* value <0.05 and |log2FC| > 1. RNA-seq data (SRA format) obtained from the SRA database were transferred to FastQ files by fastq. The quality of raw sequenced reads was assessed using FastQC. Trim Galore was adopted to trim paired-end sequencing reads with a base-call q 25, phred score <30, read length <36 and to remove Illumina adapter sequences (Cutadapt v3.4). Trimmed reads were mapped to the *Homo sapiens* (GRCh38) or *Mus musculus* reference genome (GRCm39) by HISAT2 software (v2.1.0) (Kim et al., 2015). On average, 95% of reads had a unique match to the reference genome. Next, mapped reads (SAM format) were transferred to BAM files and sorted with SAMTools (v1.7) (Li et al., 2009). For quantification, featureCounts (v2.0.1) was used to obtain raw count data using the ENSEMBL reference (release 104) (Liao et al., 2014). For biological duplicate samples (n ≥ 2), DESeq2 in R was applied to perform a differential expression analysis (Love et al., 2014), while the ‘edgeR’ package was utilized for nonbiological duplicate samples (n = 1) based on raw count data (Robinson et al., 2010). The DEGs identified were visualized by the “ggplot2” and “pheatmap” packages in R. Overlapping DEGs from different databases were displayed with Venn diagrams.

### Enrichment analysis

GO, KEGG and GSEA analyses using the “clusterProfiler” package in R software were adopted to reveal the potential functions of DEGs with the strict cutoff of *p* < 0.01 and FDR <0.05 (Yu et al., 2012). The visualization of GSEA results was produced *via* the “enrichplot” package in R.

### PPI network analysis, MCODE cluster modules and hub gene identification

The PPI network of DEGs visualized by Cytoscape was constructed by the online tool STRING (https://string-db.org/) with a combined score ≥0.4. Then, the MCODE plug-in in Cytoscape was used for cluster analysis (Bader and Hogue, 2003), with the following parameters: degree cutoff = 2, k-core = 2, node score cutoff = 0.2, and max depth = 100. Subsequently, the “cytoHubba” plug-in in Cytoscape was used to identify hub genes in the PPI network based on five algorithms, including Degree, MCC, MNC, EPC, and Closeness (Chin et al., 2014).

### Target miRNA prediction and miRNA-mRNA network construction

MiRNA-mRNA interactions confirmed by luciferase reporter assay were identified by the multiMiR R package (Ru et al., 2014). A miRNA-mRNA network was constructed by Cytoscape.

### Cell culture

The human bone marrow-derived mesenchymal stem cells (hMSCs) were purchased from the Cell Bank of the Chinese Academy of Sciences (SCSP-405; Shanghai, China) and cultured in complete medium containing 500 ml NutriStem®MSC XF Basal Medium (Biological Industries, Israel), 3 ml NutriStem^®^ MSCXF Supplement (Biological Industries, Israel) and 5 ml penicillin and streptomycin (ThermoFisher Scientific, Waltham, MA, United States) in an atmosphere of 5% CO_2_ and 95% humidity at 37°C. The culture medium was changed every 2 days. Cells beyond passage 10 were not allowed for experiments. For osteoblast differentiation, hMSCs at an appropriate confluency were induced by the addition of 100 nM dexamethasone, 10 mM β-glycerophosphate, and 50 μg/ml ascorbic acid. Cell experiments were repeated three times (n = 3).

### 2D clinorotation

A 2D clinostat (developed by the China Astronaut Research and Training Center, Beijing, China) was used for simulating microgravity condition *in vitro*. Briefly, hMSCs were seeded in a 25-cm^2^ culture flask at a density of 5 × 10^5^ cells. After cell adherence, the flask was filled with complete medium, ensuring air bubbles were removed. Then, the culture flask was rotated around the horizontal axis at 24 rpm for 72 h while being fixed to the 2D clinostat. But the CON groups were placed in a similar incubator without clinorotation at the same time.

### RNA extraction and quantitative real-time polymerase chain reaction (qRT-PCR) analysis

The total RNA of cells was isolated using RNAiso Plus (TaKaRa, Dalian, China). A Prime Script™ RT Master Mix Kit (TaKaRa, Dalian, China) was used to reverse-transcribe total RNA to complementary DNA (cDNA) by the following procedure: 37°C for 15 min, 85°C for 5 s, and 4°C for holding. For miRNA expression analysis, miRNA was reverse-transcribed using a Mir-X miRNA First-Strand Synthesis Kit (Clontech, Palo Alto, CA, United States) by the following procedure: incubation at 37°C for 1 h, termination at 85°C for 5 min, and holding at 4°C. qRT-PCR reactions were performed using a SYBR^®^ Premix Ex Taq™ II Kit (TaKaRa, Dalian, China) and a CFX96 real-time PCR detection system (Bio-Rad Laboratories, Hercules, CA, United States). Quantification of gene expression was performed with the comparative threshold cycle (ΔΔCT) method, and the expression of mRNAs was normalized to endogenous GAPDH and the expression of miRNA was normalized to endogenous U6. The primer sequences are shown in Supplementary Table S2.

### Cell transfection

Cells were transfected with the miRNA mimic (40 nM), siRNAs (80 nM) specific for hub genes or their corresponding negative controls (GenePharma, Shanghai, China) using a Lipofectamine 3000 kit (Thermo Fisher Scientific, Waltham, MA, United States) following the manufacturer’s instructions. The sequences of the siRNAs are shown in Supplementary Table S3.

### Protein isolation and western blotting analysis

The experiments were performed as previously reported (Wang et al., 2020). Briefly, total protein was extracted from cells and was quantitated. After electrophoresis, the separated proteins were transferred to polyvinylidene difluoride (PVDF) membranes. Then, PVDF membranes were blocked with 5% nonfat milk in TBST and incubated with primary antibodies specific for RUNX2 (1:1000, Cell Signaling Technology #12556S, United States), BGLAP (1:2000, Abcam ab93876, United States), GAPDH (1:5000, Proteintech 60004-1-Ig, United States). Next, PVDF membranes were incubated with peroxidase-conjugated goat anti-rabbit IgG or goat anti-mouse IgG (1:5000, ZSGB-BIO, Beijing, China) and were visualized by chemiluminescence reagent (Millipore, Billerica, MA, United States). GAPDH served as the reference gene. The relative quantity of protein expression was analyzed with the ImageJ software.

### Statistical analysis

qRT-PCR and western blot data are expressed as the means ± SDs, and were analyzed using SPSS Statistics 22.0. Two-group comparisons were performed using Student’s t test and multiple group comparisons were analyzed by one-way ANOVA followed by the LSD post hoc test. *p* < 0.05 was considered significant.

## Results

### Analysis of RNA expression patterns in *Homo sapiens*

#### Identification of differentially expressed genes (DEGs) in the GSE100900 dataset

**FIGURE 1:**
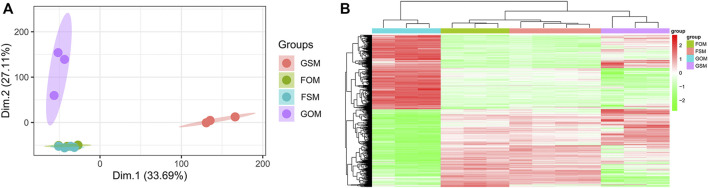
Identification of DEGs in the GSE100900 dataset **(A)** PCA for gene expression **(B)** Heatmap and hierarchical clustering of 1814 microgravity-sensitive genes in osteogenic medium (FOMvsGOM).

The GSE100900 dataset, which includes three in-flight hMSCs cultured in osteogenic medium (FOM), four in-flight hMSCs cultured in standard medium (FSM), three ground-based hMSCs cultured in osteogenic medium (GOM) and three ground-based hMSCs cultured in standard medium (GSM), was first selected for analysis. Principal component analysis (PCA) for gene expression in the GSE100900 dataset elucidated that FOM showed significant distinction with GSM and GOM, but not FSM subgroups (Figure 1A). Also, microgravity-sensitive genes in osteogenic medium (FOMvsGOM) or standard medium (FSMvsGSM), osteogenesis-sensitive genes on ground (GOMvsGSM) or in space (FOMvsFSM) were visualized by a heatmap and volcano plots (Figure 1B, Supplementary Figures S1A-D).

### Enrichment analysis of GSE100930 dataset

**FIGURE 2:**
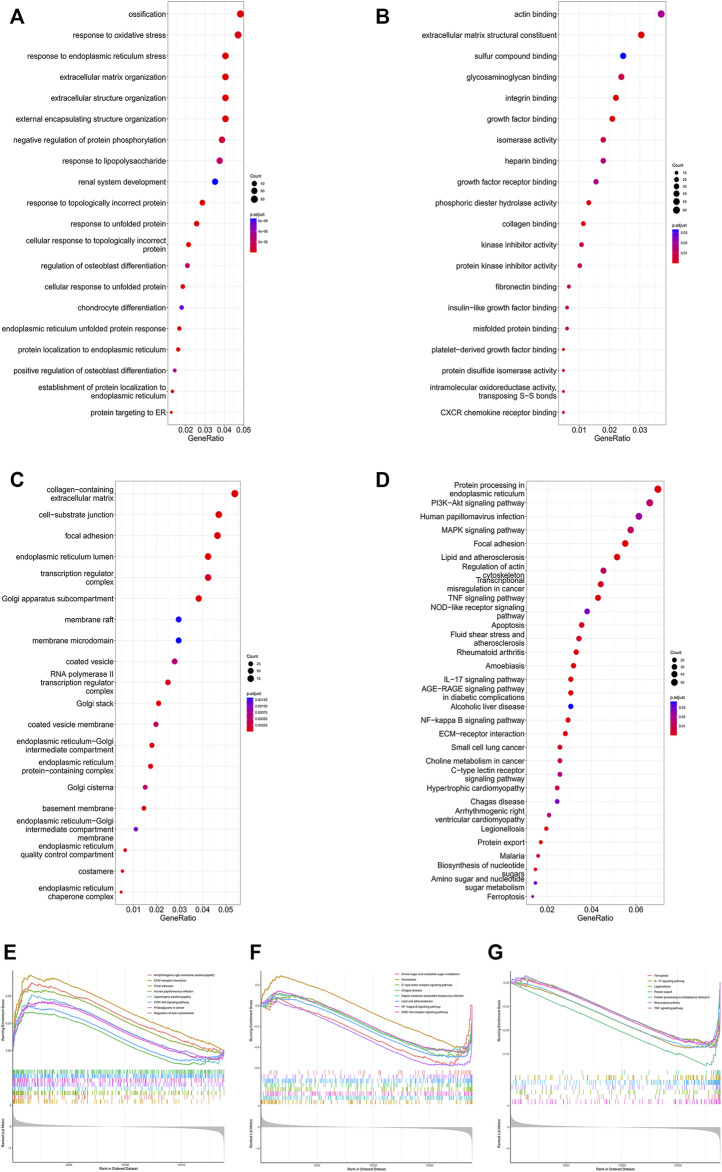
Enrichment analysis of DEGs in the GSE100900 dataset The top 30 BPs **(A)**, MFs **(B)** and CCs **(C)** in the GO analysis of microgravity-sensitive genes in osteogenic medium (FOMvsGOM) are shown **(D)** KEGG analysis of microgravity-sensitive genes in osteogenic medium (FOMvsGOM). The size of the circle indicates the gene count. The color of the circles represents the adjusted *p* value **(E–G)** The 23 terms of GSEA that overlapped with the KEGG results are presented.

To further identify microgravity-sensitive genes during osteogenic differentiation, we screened out 1814 DEGs that were markedly changed after exposure to real microgravity conditions for 14 days for GO analysis, which demonstrated that three biological progresses (BPs), three cellular components (CC) and three molecular functions (MF) were the most enriched processes in the progression of microgravity-induced bone loss, including extracellular matrix organization (BP), extracellular structure organization (BP), response to endoplasmic reticulum stress (BP), collagen-containing extracellular matrix (CC), focal adhesion (CC), cell-substrate junction (CC), actin binding (MF), phosphoric ester hydrolase activity (MF) and extracellular matrix structural constituent (MF) (Figures 2A–C). The KEGG pathway analysis results indicated that the 1814 DEGs were mainly distributed in 31 KEGG pathways, including protein processing in the endoplasmic reticulum, the PI3K-Akt signaling pathway, and the MAPK signaling pathway (Figure 2D). GSEA of all genes showed that 66 pathways were involved, and the 23 that overlapped with the KEGG results are visualized in Figures 2E–G.

### Eleven hub genes and three essential gene cluster modules were identified in the PPI network of DEGs

**FIGURE 3:**
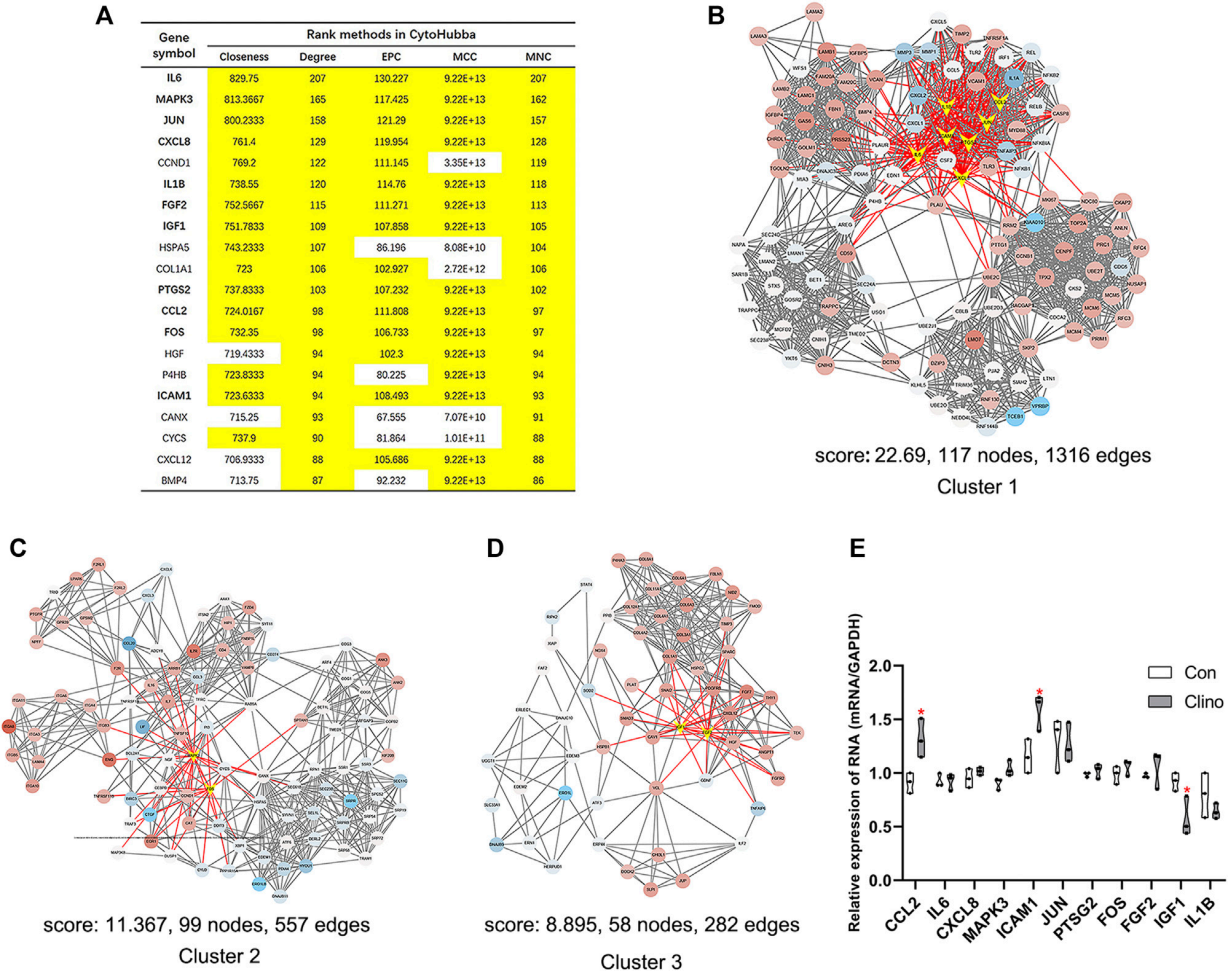
Identification of hub genes and essential gene cluster modules in the PPI network of DEGs in the GSE100900 dataset **(A)** The hub genes identified by five algorithms, including degree, MCC, MNC, EPC, and closeness. Those marked in yellow indicate that their ratings rank in the top 20 of their respective algorithms **(B–D)** The top three modules identified by MCODE. The V-shape in yellow indicates hub genes **(E)** qRT-PCR for the expression of 11 hub genes in hMSCs under clinorotation-microgravity condition for 72 h. Data are the mean ± SD. *p < 0.05, **p < 0.01.

We constructed the interaction network by String (https://string-db.org/) with a required interaction score >0.4 to further demonstrate the interactions among the 1814 DEGs. Consequently, 1662 nodes and 13,805 edges were determined and visualized by Cytoscape (Supplementary Figure S2). Next, the plug-in “cytoHubba” was used to identify hub genes based on five algorithms, including Degree, Maximal Clique Centrality (MCC), Maximum Neighborhood Component (MNC), Edge Percolated Component (EPC) and Closeness (Chin et al., 2014). The results showed that 11 genes were regarded as hub genes by intersecting the top 20 genes from the five algorithms (Figure 3A). These results indicate that these genes are the most important genes in the PPI network and may play essential roles in the pathogenesis of microgravity-induced bone loss. Moreover, we identified 34 gene cluster modules by the plug-in MCODE in this network, and three modules with high cluster scores are shown in Figures 3B–D.

### Eleven hub genes showed diverse expression patterns in hMSCs under clinorotation-microgravity condition for 72 h

2D clinorotation is a ground-based facility for simulating microgravity condition, and our studies have demonstrated that clinorotation-microgravity remarkablely disturbs the dynamic balance of bone remodeling (Hu et al., 2015; Sun et al., 2015; Wang et al., 2020). Therefore, the expression of 11 hub genes in hMSCs under clinorotation-microgravity condition for 72 h was further validated by qRT-PCR. The results showed that only the expression of CCL2, ICAM1 and IGF1 markedly changed in the clinorotation-microgravity group (Figure 3E), suggesting that these genes are worthy of further study.

### Onboard and RPM shared some common biological functions

**FIGURE 4:**
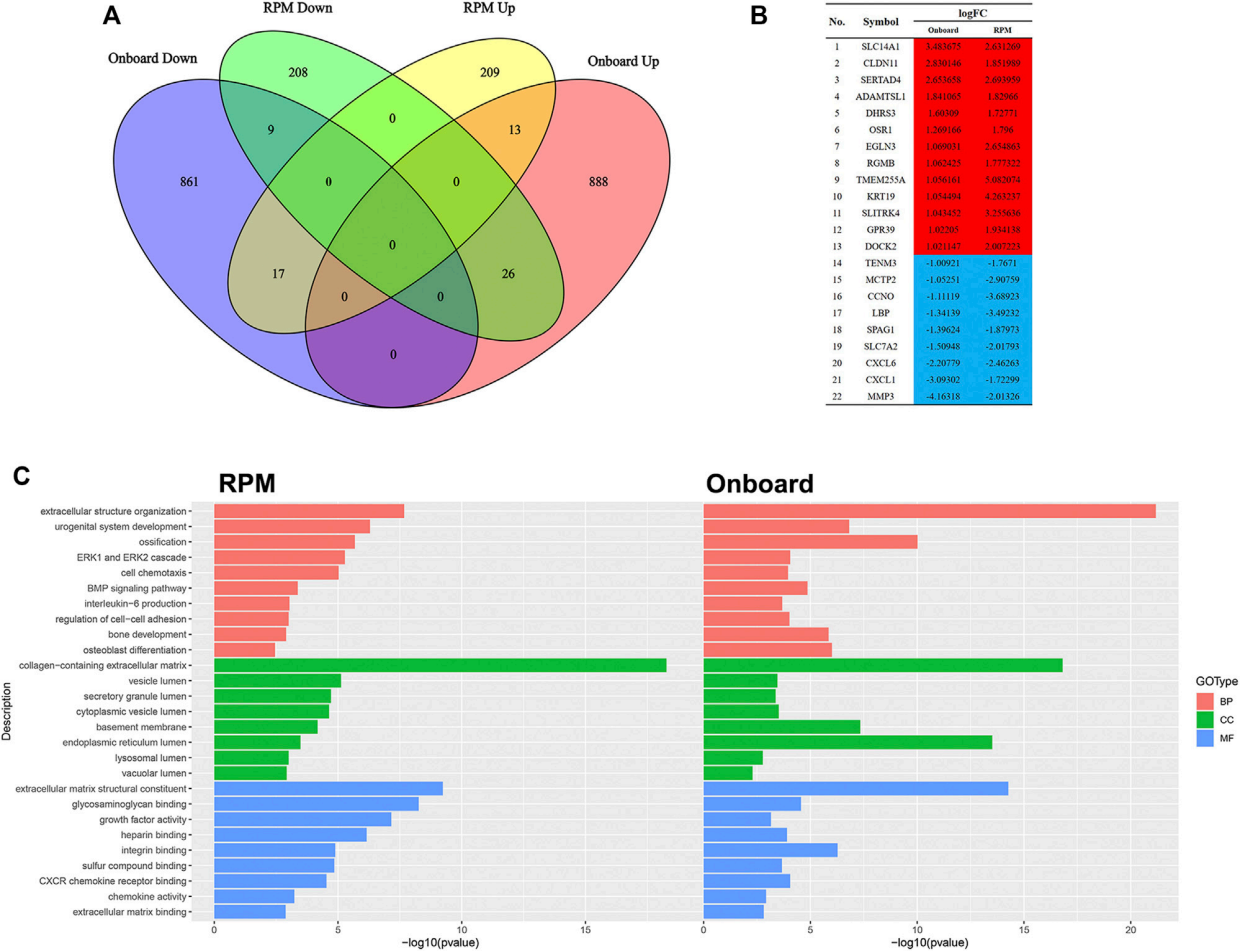
Comparison between Onboard and RPM **(A)** Venn diagrams of microgravity-sensitive genes in osteogenic medium from the Onboard (GSE100930) and RPM (GSE114117) datasets **(B)** Overlapping microgravity-sensitive genes in osteogenic medium from the Onboard and RPM databases. Red indicates upregulated genes, and blue indicates downregulated genes **(C)** Overlapping GO analysis results from the Onboard and RPM databases.

Since RPM, a ground-based microgravity simulator, and real microgravity work in distinct modes, it is necessary to compare their RNA expression patterns. The GSE114117 dataset contains seven samples of hBMSCs osteogenically induced for 0, 2, 7 and 14 days under normal gravity or RPM (Li et al., 2019). Considering that microgravity duration is a vital factor, we selected two hBMSC samples osteogenically induced for 14 days, which is consistent with the microgravity duration in the GSE100930 dataset, to explore the interactions between real microgravity and the RPM model. The comparison found 22 genes that changed in the same manner in the real microgravity and RPM models (Figures 4A, B). GO and KEGG analysis of DEGs in RPM showed that they shared 10 BPs, 8 CCs and 9 MFs with the results from real microgravity (Figure 4C). These results demonstrate that the two methods could produce relatively different results, but we still captured some common biological phenomena related to bone remodeling.

### Five miRNA-mRNA axes were regarded as crucial factors in microgravity-induced bone loss

**FIGURE 5:**
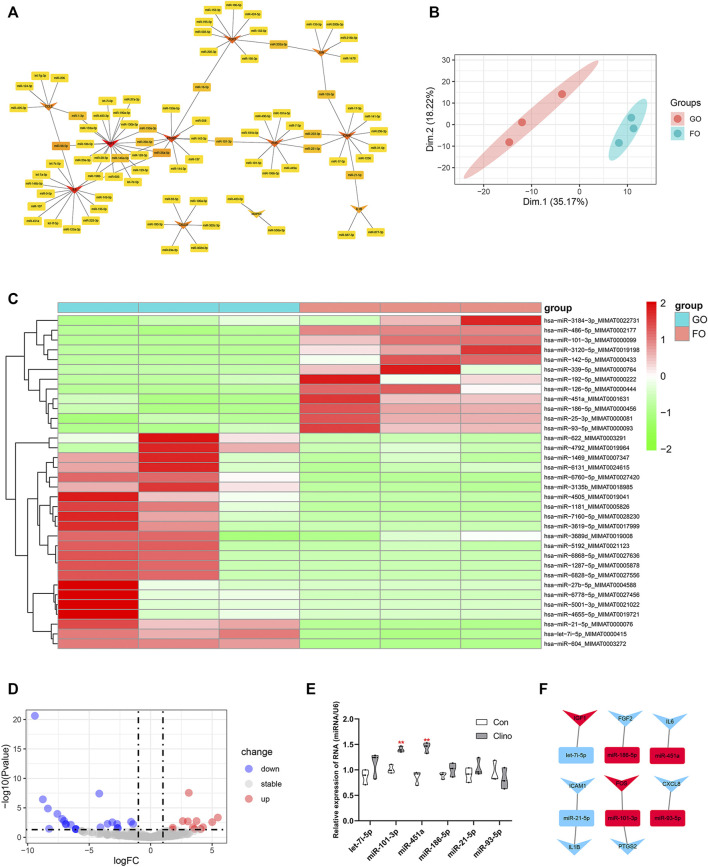
Identification of crucial miRNA-mRNA axes in microgravity-induced bone loss **(A)** Coexpressed network of hub genes and their target miRNAs **(B)** PCA for miRNA expression in the GSE100932 dataset **(C)** Heatmap and hierarchical clustering of differentially expressed miRNAs (DEMs) **(D)** Volcano plot of DEMs **(E)** qRT-PCR for the expression of six miRNAs in hMSCs under clinorotation-microgravity condition for 72 h. Data are the mean ± SD. *p < 0.05, **p < 0.01 **(F)** The interactions between hub genes and microgravity-sensitive miRNAs.

The target miRNAs of 11 hub genes that were confirmed by luciferase reporter assay were identified by the R package multiMiR (Ru et al., 2014). As a result, a coexpression network of 11 hub genes and 82 target miRNAs comprising 93 nodes and 97 edges was constructed by Cytoscape (Figure 5A). To further verify microgravity-sensitive miRNAs, we interacted 82 target miRNAs with the data from GSE100932 dataset, which is a miRNA-seq dataset from exosomes in hBMSCs osteogenically induced for 14 days under normal gravity or real microgravity environment (Silvia Bradamante et al., 2018). PCA showed significant distinctions between the FOM and GOM subgroups (Figure 5B). Visualization of 22 downregulated miRNAs and 12 upregulated miRNAs was conducted using a heatmap and a volcano plot (Figures 5C, D). Ultimately, we found that six miRNAs of the 82 target miRNAs showed significant changes under real microgravity conditions. Also, miR-101-3p and miR-451a were markedly upregulated in hMSCs under clinorotation-microgravity condition for 72 h (Figure 5E). Next, the interactions between hub genes and microgravity-sensitive miRNAs were visualized (Figure 5F).

### ICAM1 and miR-451a affected the osteogenesis of hMSCs under clinorotation-microgravity condition

**FIGURE 6:**
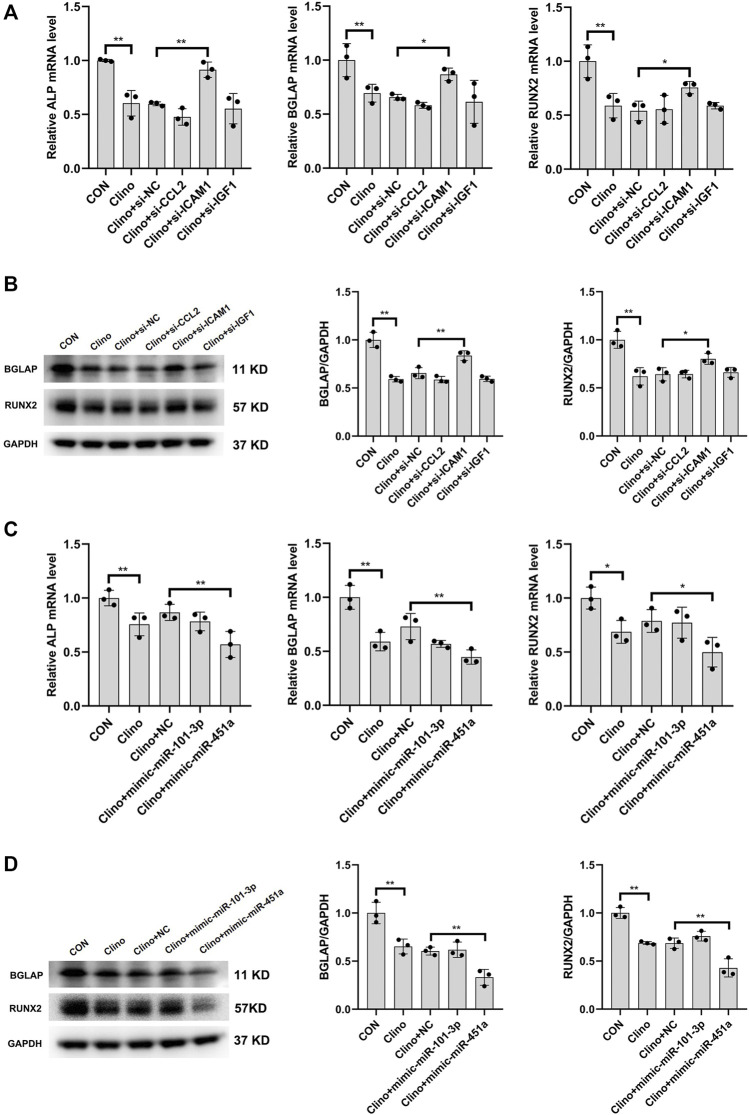
The effects of the identified clinorotation-sensitive genes and miRNAs on osteogenesis of hMSCs under clinorotation condition hMSCs were transfected with siRNA-NC or siRNA–genes and cultured in osteogenic medium under clinorotation condition for 72 h, and were then subjected to qRT-PCR analysis of ALP, BGLAP and Runx2 mRNA levels **(A)**, western blot analysis of BGLAP and Runx2 protein expression **(B)** (n = 3). hMSCs were transfected with mimic-miRNA-NC or mimic-miRNA and cultured in osteogenic medium under clinorotation condition for 72 h, and were then subjected to qRT-PCR analysis of ALP, BGLAP and Runx2 mRNA levels **(C)**, western blot analysis of BGLAP and Runx2 protein expression **(D)** (n = 3). All data are the mean ± SD. *p < 0.05, **p < 0.01.

To assess the effects of the identified clinorotation-sensitive genes and miRNAs on osteogenesis, hMSCs were transfected with siRNA-gene or mimic-miRNA for 12 h, and were then cultured in osteogenic medium under clinorotation-unloading condition for 72 h. These results showed that si-ICAM1 attenuated the reduction in the mRNA levels of osteogenic marker genes, including ALP, BGLAP and RUNX2 induced by clinorotation and the protein expression levels of BGLAP and RUNX2 were also attenuated in si-ICAM1 hMSCs (Figures 6A, B). On the contrast, mimic-miR-451a further aggravated the reduction in the mRNA levels of ALP, BGLAP, RUNX2 and the protein expression levels of BGLAP, RUNX2 induced by clinorotation (Figures 6C, D).

### Analysis of RNA expression patterns in mice

#### Identification of differentially expressed genes in SRP276872

**FIGURE 7:**
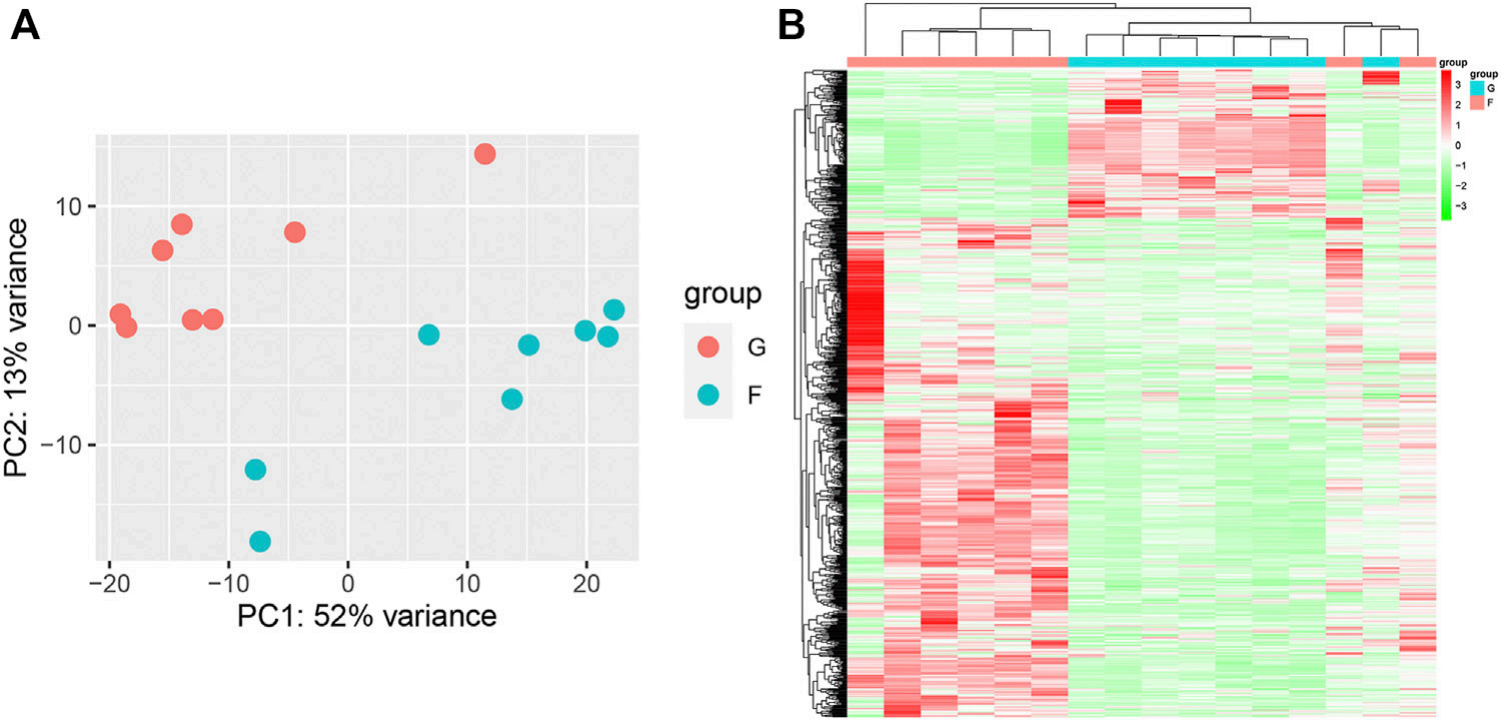
Identification of DEGs in the SRP276872 dataset **(A)** PCA for gene expression **(B)** Heatmap and hierarchical clustering of DEGs.

PCA showed remarkable differences between the flight (F) and ground (G) subgroups in the SRP276872 dataset (Supplementary Figure S3A). Then, 838 DEGs (192 downregulated genes and 646 upregulated genes) in the femurs of mice as a result of exposure to microgravity for 33 days were identified and visualized by a heatmap and a volcano plot (Figures 7A, B).

### Enrichment analysis of SRP276872 dataset

**FIGURE 8:**
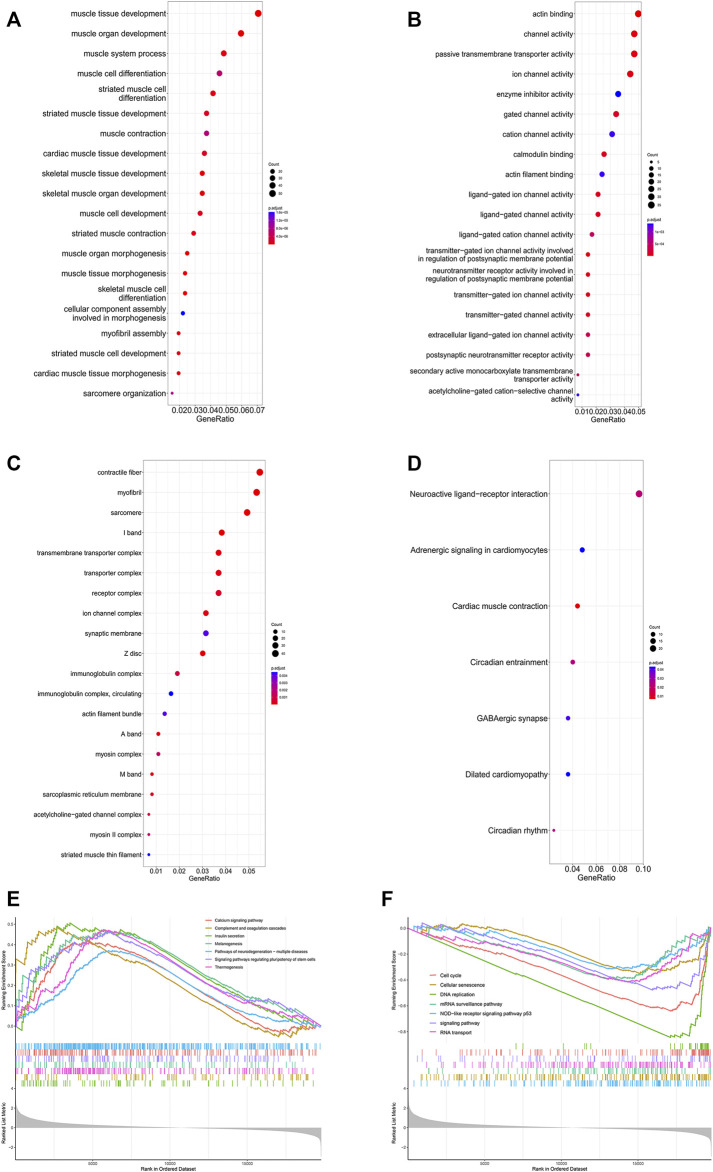
Enrichment analysis of DEGs in the SRP276872 dataset The top 30 BPs **(A)**, MFs **(B)** and CCs **(C)** in the GO analysis of DEGs are shown **(D)** KEGG analysis of DEGs. The size of the circle indicates the gene count. The color of the circles represents the adjusted *p* value **(E) (F)** Visualization of 14 of the 51 GSEA terms.

The GO analysis results are shown in Figures 8A–C. The top three BPs were muscle tissue development, striated muscle tissue development and muscle organ development. Contractile fiber, myofibril and actin cytoskeleton were the top three enriched CCs. In addition, the MF GO terms mainly contained actin binding, channel activity and passive transmembrane transporter activity. In addition, the KEGG analysis indicated seven enriched pathways (Figure 8D). Four of these pathways, including neuroactive ligand-receptor interaction, cardiac muscle contraction, adrenergic signaling in cardiomyocytes and dilated cardiomyopathy, were also enriched in GSEA. There were 51 pathways involved in GSEA in total, and 14 of them are shown in Figures 8E, F.

### PPI network analysis, MCODE cluster modules and hub gene identification

**FIGURE 9:**
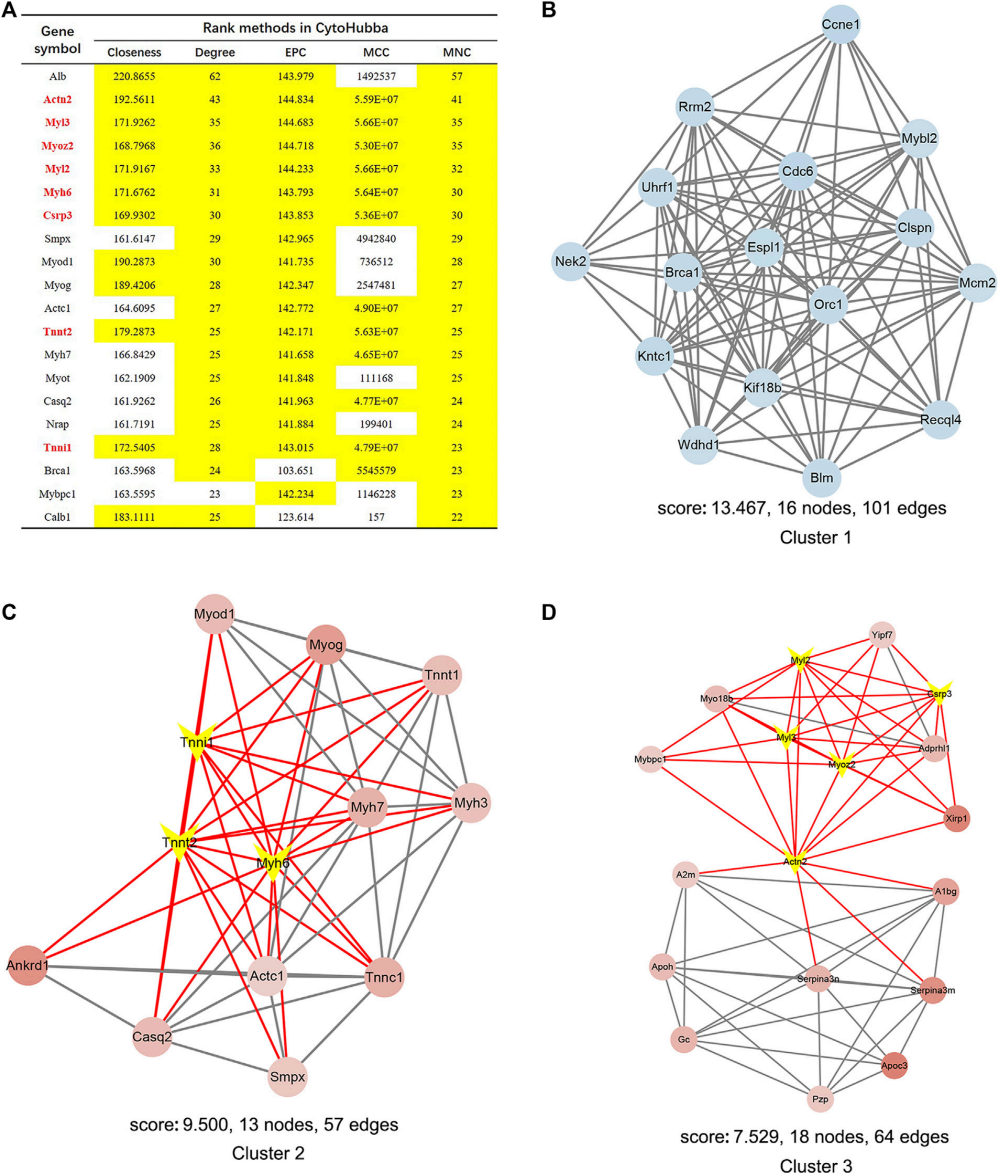
Identification of hub genes and essential gene cluster modules in the PPI network of DEGs in the SRP276872 dataset **(A)** The hub genes identified by five algorithms, including degree, MCC, MNC, EPC, and closeness. Those marked yellow indicate that their ratings rank in the top 20 of their respective algorithms **(B–D)**. The top three modules identified by MCODE. The V-shape in yellow indicates hub genes.

PPI network analysis by String demonstrated that the interaction network of the 838 DEGs included 510 nodes and 1549 edges visualized by Cytoscape (Supplementary Figure S3B). Next, the eight hub genes in this network are highlighted in Figure 9A. MCODE identified 27 gene cluster modules, and three modules with high cluster scores are shown in Figures 9B–D.

### Comparison with microgravity simulators on the ground

**FIGURE 10:**
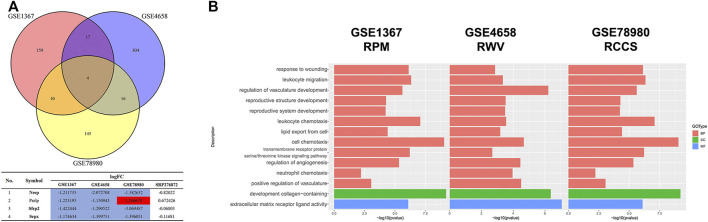
Comparison among the results from ground-based microgravity simulators in mice **(A)** Venn diagrams of the DEGs from these three datasets **(B)** Overlapping GO analysis results from these three datasets.

Since the microgravity simulators on the ground and real microgravity work through distinct modes, it is imperative to compare the data produced by each model. In total, three datasets, GSE1367, GSE4658 and GSE78980, were chosen for further analysis. The DEGs from each dataset are illustrated in volcano plots (Supplementary Figure S4A-C). Crosstalk analysis elucidated that only four genes were differentially expressed in the three datasets, but none of these genes were significantly changed in the SRP276872 dataset (Figure 10A). GO analysis showed that these three databases shared 12 BPs, 1 CC and 1 MF (Figure 10B).

## Discussion

Bone remodeling is a process that relies on the dynamic balance between bone formation and bone resorption (Long, 2011). Under microgravity conditions, skeletal unloading can induce severe disuse osteopenia, which is a complicated pathological process regulated by a variety of factors (Kostenuik et al., 1999). Although transcriptome profiling has been widely utilized to explore the mechanism of microgravity-induced bone loss, scant consistent conclusions are universally accepted (Dai et al., 2007; Zhang et al., 2018; Li et al., 2019; Lee et al., 2020). In this study, we analyzed and compared eight transcriptome datasets related to microgravity-induced bone loss but struggled to find common results between these different studies. Collectively, we identified 11 hub genes and some vital miRNA-mRNA interactions under real microgravity conditions, making efforts to shed light on the study of microgravity-induced bone loss.

Multiple studies have illustrated that microgravity can affect the functions of bone-related cells by different molecular mechanisms. For instance, under microgravity conditions, the potential of osteoprogenitor cells to mature into osteoblasts is impaired, and the proliferation and differentiation of mature osteoblasts are inhibited, all of which are important causes of microgravity-induced bone loss (Caillot-Augusseau et al., 1998; Zayzafoon et al., 2004; Dai et al., 2013). Osteocytes act as mechanosensory cells that are essential for the initiation of bone remodeling, but microgravity disturbs this process by altering the cytoskeletal structure and interfering with interstitial fluid around the lacune-tubule system of osteocytes (Arfat et al., 2014). Moreover, spaceflight results elucidated that the maturity and activity of osteoclasts on Foton-M3 were significantly enhanced and that the number of trap-positive osteoclasts in cancellous bone of C57BL/6J mice on STS-131 was also increased (Nabavi et al., 2011; Blaber et al., 2013). The present study found that the cell phenotype of hMSCs after exposure to real microgravity for 14 days changed significantly, as indicated by 1814 DEGs. Moreover, these DEGs were enriched in extracellular matrix organization (BP), collagen-containing extracellular matrix (CC) and ECM-receptor interaction pathways (KEGG and GSEA). These molecular mechanisms are vital for bone remodeling because the extracellular matrix has been verified to act as a storage of calcium and phosphate ions, providing remarkable mechanical properties for bones (Ansari et al., 2021).

The 11 identified hub genes play crucial roles in bone remodeling regulated by mechanical force. Harris attributed impaired osteoblast function in orbital spaceflight to reduced mRNA expression of IL-6, a myokine that promotes bone formation and protects against bone resorption on Earth (Smith, 2018). MAPK3, also named ERK1, is a member of the ERK signaling pathway that enhances the force-induced OPG upregulation and the force-suppressed functions of osteoclast-like cells (Kook et al., 2009). JUN and FOS are both AP-1 transcription factor subunits that are essential for AP-1 activation and vital factors of the RANKL–RANK signaling cascade. Studies have demonstrated that RCCS promotes osteoclast differentiation and bone resorption by upregulating the expression of JUN and FOS (Sambandam et al., 2010; Ethiraj et al., 2020). Additionally, the differentiation of stromal osteoblastic cells was stimulated by an increase in RANKL mRNA expression and protein production induced by CXCL8 (IL-8), a force-sensitive factor, validating the positive role of CXCL8 in osteoclast formation (Bendre et al., 2003; Kapoor et al., 2014). IL-1, which functions as another inflammatory signal, has also been reported to respond to mechanical load and regulate bone remodeling in osteoblasts (Maeda et al., 2015). Moreover, FGF2 alleviated the HLU-decreased osteogenic potential of MSCs by activating Runx2 phosphorylation and the RWV-inhibited osteogenic differentiation of MSCs by combining with SB203580 and BMP-2 (Zheng et al., 2007; Qian et al., 2014). Additionally, microgravity in spaceflight or HLU significantly increased the mRNA and protein levels of IGF-1, which mediated the effects of gravity on Cbfa1 activity *via* PI3K signaling (Bikle et al., 1994; Dai et al., 2014; Smith et al., 2015). PTGS2, also known as COX2 and a downstream target of NOS2, promoted proliferation and inhibited differentiation in osteoblasts subjected to RWV (Veeriah et al., 2016). Notably, mechanical loading motivated the migration of endosteal progenitors by upregulating the expression of CCL2, and neutralizing CCL2 partially reversed the increased bone formation in mice after 4 weeks running (Yao et al., 2021). Interestingly, the force-induced mRNA increases of CCL2 could be significantly abrogated by neutralization of IL-1 (Maeda et al., 2015). Similarly, as the conventional molecule of cell adhesion that initiates biomechanical signaling, ICAM-1 expression was reduced on SJ-10 satellite (Lü et al., 2019). The above results indicated that all of the 11 hub genes are involved in the bone remodeling process regulated by mechanical force *via* a variety of mechanisms, but only CCL2, ICAM1 and IGF1 were clinorotation-sensitive validated by qRT-PCR, informing us that they are worthy of further study as a targeted therapy for microgravity-induced bone loss.

Multiple miRNA-mRNA axes have been confirmed to play regulatory roles in the proliferation, differentiation and apoptosis of various bone cells by inhibiting protein translation or degrading the mRNA of target genes during microgravity. For example, bone-targeted miR-214 alleviated HLU-induced bone loss *via* the miR-214/ATF axis, and inhibition of miR-208a-3p expression by injecting antago-miR-208a-3p *in vivo* abolished impaired bone formation through targeting ACVR1 in HLU mice (Wang et al., 2013; Arfat et al., 2018). The present study also identified some miRNA-mRNA axes, such as let-7i-5p-IGF1, miR-101-3p-PTGS2, miR-186-5p-FGF2, miR-451a-IL6 and miR-93-5p-CXCL8. Although the roles that let-7i-5p and IGF1 play in osteogenesis have been elucidated, how the let-7i-5p-IGF1 axis affects osteogenesis, especially microgravity-induced bone loss, remains to be validated (Zhang et al., 2021). MiR-101-3p is an osteogenic differentiation promoter that targets PLAP-1, DDK1 and EZH2 (Li et al., 2012; Wang et al., 2016; Xiang et al., 2020). Moreover, the interaction between miR-101-3p and PTGS2 has also been demonstrated in colon cancer, gastric cancer and esophageal squamous cell carcinoma but not in bone remodeling (Strillacci et al., 2009; Wang et al., 2010; Gong et al., 2016). In contrast, miR-186-5p and miRNA-93-5p were reported to be osteogenic differentiation inhibitors *via* various target genes (Zhang et al., 2017; Xu et al., 2019; Xiao et al., 2020; Li et al., 2021). Interestingly, it was reported that miR-451a induced osteoblast differentiation and mineralization *in vitro* and significantly reversed ovariectomy-induced bone loss *in vivo* (Karvande et al., 2018). However, miR-451a-knockout (KO) mice showed redundant bone formation in another study (Lu et al., 2019). This contradiction requires further exploration. In total, the roles these miRNAs and mRNAs serve in bone remodeling have been partly demonstrated. However, their roles and miRNA-mRNA interactions in microgravity-induced bone loss remain uncertain and require further study.

Admittedly, the best way to explore the mechanisms of microgravity-induced bone loss is to conduct studies on humans in space directly, but the limited samples and huge cost discourage many researchers from carrying out meaningful studies. The aim of our study was to compare RNA expression patterns in different microgravity-induced bone loss models, including ground-based and real microgravity models. Unfortunately, the wide variation between these models makes it difficult to find consistent hub genes and molecular mechanisms. Consequently, more reliable microgravity simulators, cell models and animal models should be developed and applied. Nevertheless, we tried to identify common genes and molecular signals mainly based on human data, but these conclusions need to be further validated. However, for the mouse data, the reason we placed special emphasis on the analysis of the SRP276872 dataset, which was exposed to real microgravity for 33 days, is because the comparison with the results from the ground-based microgravity simulators showed a less common phenomenon. Therefore, there is an extensive and arduous method to elucidate the mechanisms of microgravity-induced bone loss, and effective targeted countermeasures remain to be explored.

## Data Availability

The original contributions presented in the study are included in the article/Supplementary Material, further inquiries can be directed to the corresponding authors.
