# Supplementary material for: Bioinformatic analysis of the RNA expression patterns in microgravity-induced bone loss
Source: Front Genet. 2022 Nov 8;13:985025. doi: 10.3389/fgene.2022.985025 (PMC9681495; doi:10.3389/fgene.2022.985025)
Supplement: Supplementary file 1 [file Table1.doc]

**Table S1. Characteristics of involved datasets**


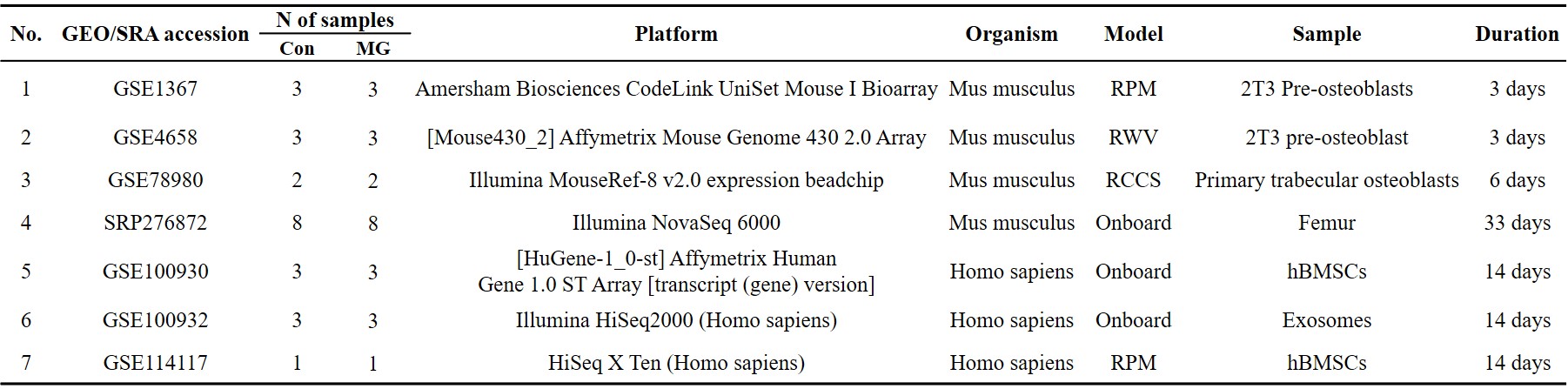


**Table S2. Primers used for qRT-PCR**

| **Gene symbol** | **Forward 5' - 3'** | **Reverse 5' - 3'** |
| --- | --- | --- |
| **GAPDH** | TGTGTCCGTCGTGGATCTGA | TTGCTGTTGAAGTCGCAGGAG |
| **ICAM1** | TGCAAGAAGATAGCCAACCAAT | GTACACGGTGAGGAAGGTTTTA |
| **FOS** | CTTCCCAGAAGAGATGTCTGTG | TGGGAACAGGAAGTCATCAAAG |
| **CCL2** | ACCAGCAGCAAGTGTCCCAAAG | TTTGCTTGTCCAGGTGGTCCATG |
| **PTGS2** | TGTCAAAACCGAGGTGTATGTA | AACGTTCCAAAATCCCTTGAAG |
| **IGF1** | AAAAATCAGCAGTCTTCCAACC | CCTGTGGGCTTGTTGAAATAAA |
| **FGF2** | CATCAAGCTACAACTTCAAGCA | CCGTAACACATTTAGAAGCCAG |
| **IL1B** | GCCAGTGAAATGATGGCTTATT | AGGAGCACTTCATCTGTTTAGG |
| **CXCL8** | AACTGAGAGTGATTGAGAGTGG | ATGAATTCTCAGCCCTCTTCAA |
| **JUN** | CAAACCTCAGCAACTTCAACC | CTGGGACTCCATGTCGATG |
| **MAPK3** | TCTGCTACTTCCTCTACCAGAT | CAGGCCGAAATCACAAATCTTA |
| **IL6** | CACTGGTCTTTTGGAGTTTGAG | GGACTTTTGTACTCATCTGCAC |
| **ALP** | ACTCTCCGAGATGGTGGTGGTG | CGTGGTCAATTCTGCCTCCTTCC |
| **BGLAP** | AGGGCAGCGAGGTAGTGAAGAG | GCCGATGTGGTCAGCCAACTC |
| **RUNX2** | AGGCAGTTCCCAAGCATTTCATCC | TGGCAGGTAGGTGTGGTAGTGAG |
| **U6** | CTCGCTTCGGCAGCACA | AACGCTTCACGAATTTGCGT |
| **hsa-let-7i-5p** | GCGTGAGGTAGTAGTTTGTGCTGTT | The 3' primer for qRT-PCR analysis of miRNAs expression is the mRQ 3' Primer supplied with the Mir-X™ miRNA First-Strand Synthesis kit (Clontech, USA). |
| **hsa-miR-101-3p** | GCAGCGCTACAGTACTGTGATAACTGA |
| **hsa-miR-186-5p** | CGTGCAAAGAATTCTCCTTTTGGGCT |
| **hsa-miR-21-5p** | AGCGCTAGCTTATCAGACTGATGTTGA |
| **hsa-miR-451a** | GCGCAAACCGTTACCATTACTGAGTT |
| **hsa-miR-93-5p** | TCAAAGTGCTGTTCGTGCAGGTAG |

**Table S3.** **siRNAs sequences**

| **Name** | **Sequence 5' - 3'** |
| --- | --- |
| **siRNA-CCL2 sense** | GCUGUUAUAACUUCACCAATT |
| **siRNA-CCL2 antisense** | UUGGUGAAGUUAUAACAGCTT |
| **siRNA-ICAM1 sense** | CAGCGGAAGAUCAAGAAAUTT |
| **siRNA-ICAM1 antisense** | AUUUCUUGAUCUUCCGCUGTT |
| **siRNA-IGF1 sense** | GUACAUUUGAAGAACGCAATT |
| **siRNA-IGF1 antisense** | UUGCGUUCUUCAAAUGUACTT |
| **siRNA-control sense** | UUCUCCGAACGUGUCACGUTT |
| **siRNA-control antisense** | ACGUGACACGUUCGGAGAATT |


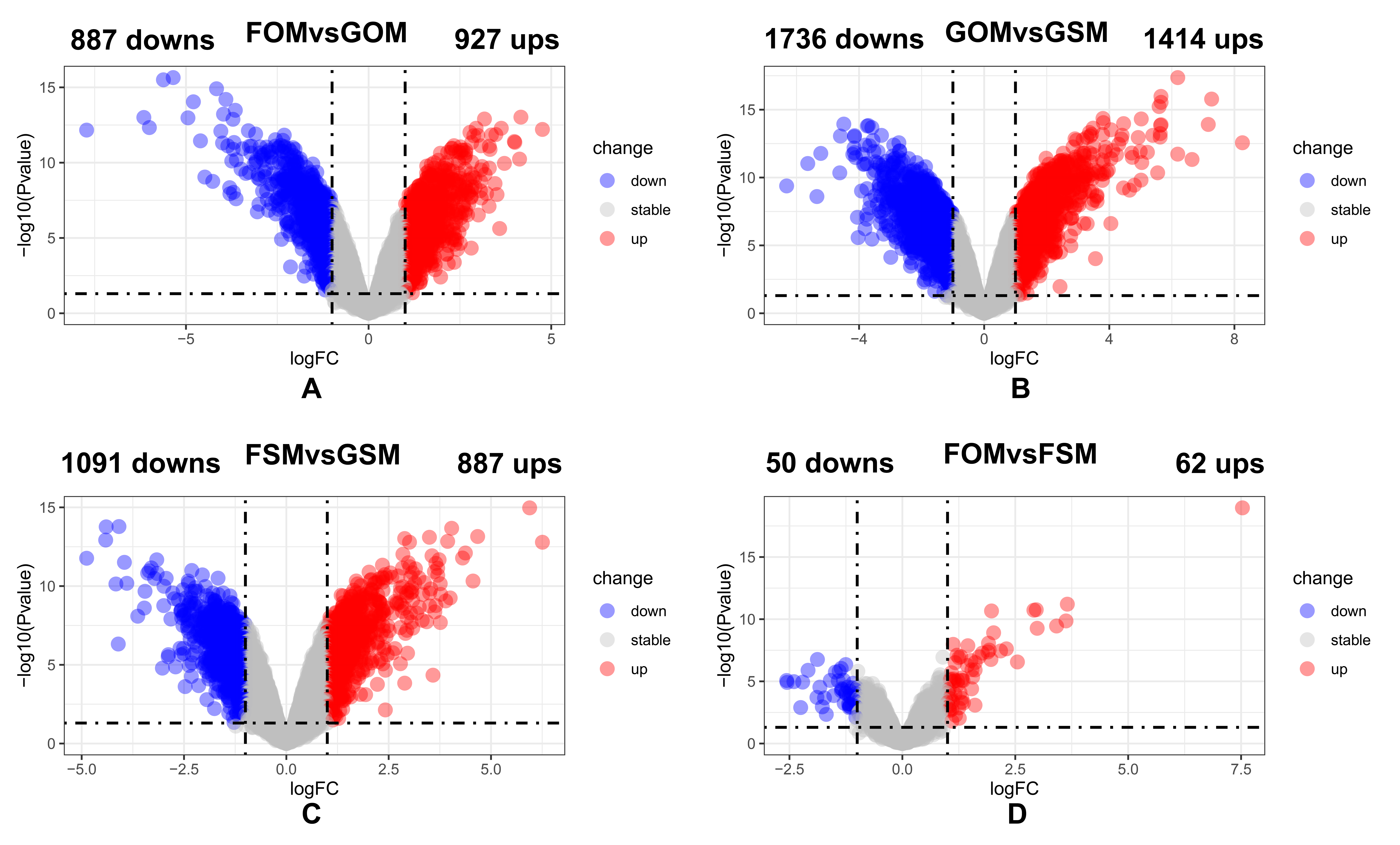


**Figure S1:** (A) Volcano plot of microgravity-sensitive genes in osteogenic medium (FOMvsGOM). (B) Volcano plot of osteogenesis-sensitive genes on ground (GOMvsGSM). (C) Volcano plot of microgravity-sensitive genes in standard medium (FSMvsGSM). (D) Volcano plot of osteogenesis-sensitive genes in space (FOMvsFSM).


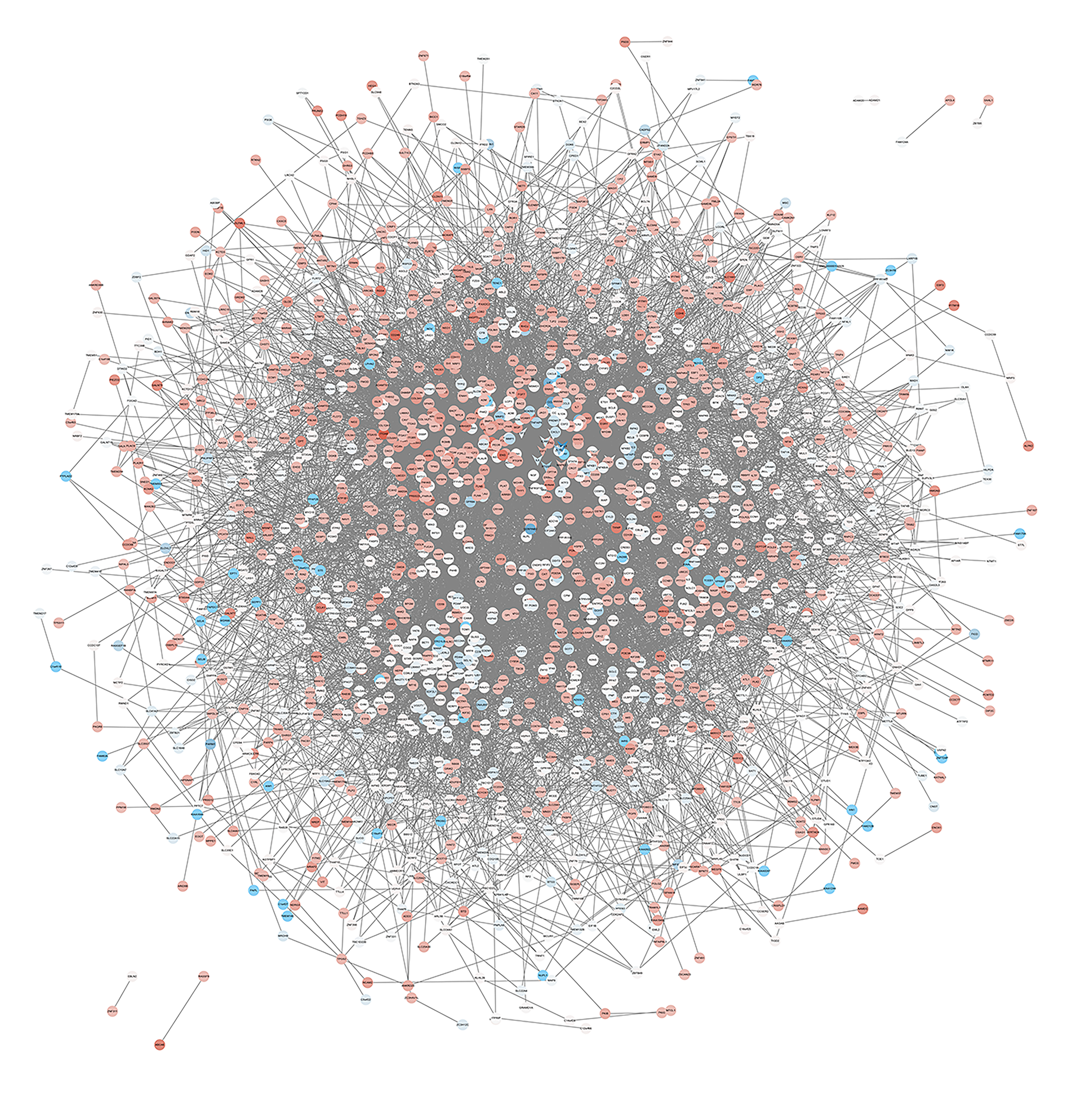


**Figure S2: PPI network via STRING in the GSE100930 dataset.** Node represents gene and edge represents PPIs. Multiples of DEG changes in real microgravity conditions compared with normal controls are indicated by different colors.





**Figure S3:** (A) Volcano plot of DEGs in the SRP276872 dataset. (B) PPI network via STRING. Node represents gene and edge represents PPIs. Multiples of DEG changes in real microgravity conditions compared with normal controls are indicated by different colors.


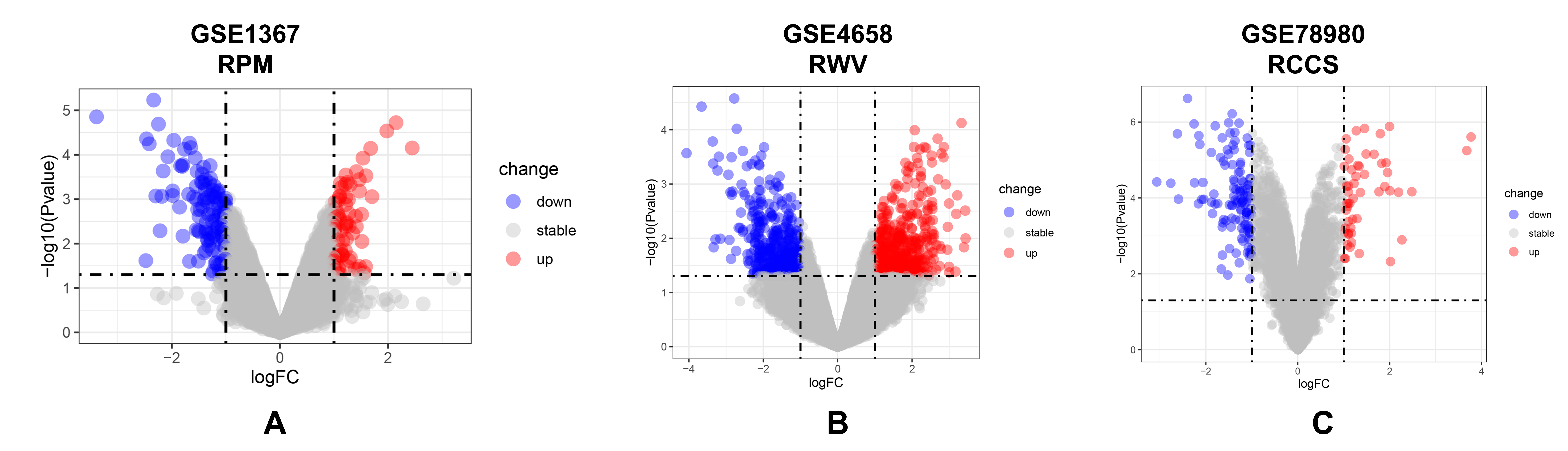
 **Figure S4:** (A)-(C) Volcano plots showing the DEGs in GSE1367, GSE4658 and GSE78980.
